# Tissue-based metabolomics reveals metabolic signatures and major metabolic pathways of gastric cancer with help of transcriptomic data from TCGA

**DOI:** 10.1042/BSR20211476

**Published:** 2021-10-04

**Authors:** Yaqin Wang, Wenchao Chen, Kun Li, Gang Wu, Wei Zhang, Peizhi Ma, Siqi Feng

**Affiliations:** 1 Department of pharmacy, Henan Provincial People’s Hospital, People’s Hospital of Zhengzhou University, School of Clinical Medicine, Henan University, Zhengzhou, Henan 450003, China; 2 Department of Gastrointestinal Surgery, Henan Provincial People's Hospital, People's Hospital of Zhengzhou University, School of Clinical Medicine, Henan University, Zhengzhou, Henan 450003, China; 3 Key Laboratory of Advanced Pharmaceutical Technology, Ministry of Education of China; Co-innovation Center of Henan Province for New Drug R&D and Preclinical Safety; School of Pharmaceutical Sciences, Zhengzhou University, Zhengzhou, Henan 450001, China

**Keywords:** Gastric cancer, Glycerophospholipid metabolism, Metabolomics, Transcriptomics

## Abstract

Purpose: The aim of the present study was to screen differential metabolites of gastric cancer (GC) and identify the key metabolic pathways of GC.

Methods: GC (*n*=28) and matched paracancerous (PC) tissues were collected, and LC-MS/MS analysis were performed to detect metabolites of GC and PC tissues. Metabolite pathways based on differential metabolites were enriched by MetaboAnalyst, and genes related to metabolite pathways were identified using the KEGGREST function of the R software package. Transcriptomics data from The Cancer Genome Atlas (TCGA) was analyzed to obtain differentially expressed genes (DEGs) of GC. Overlapping genes were acquired from metabonimics and transcriptomics data. Pathway enrichment analysis was performed using String. The protein expression of genes was validated by the Human Protein Atlas (HPA) database.

Results: A total of 325 key metabolites were identified, 111 of which were differentially expressed between the GC and PC groups. Seven metabolite pathways enriched by MetaboAnalyst were chosen, and 361 genes were identified by KEGGREST. A total of 2831 DEGs were identified from the TCGA cohort. Of these, 1317 were down-regulated, and 1636 were up-regulated. Twenty-two overlapping genes were identified between genes related to metabolism and DEGs. Glycerophospholipid (GPL) metabolism is likely associated with GC, of which AGPAT9 and ETNPPL showed lower expressed in GC tissues.

Conclusions: We investigated the tissue-based metabolomics profile of GC, and several differential metabolites were identified. GPL metabolism may affect on progression of GC.

## Introduction

Gastric cancer (GC) is one of the most common malignant tumors and ranks fifth as the cause of death among 36 cancers in the world [1]. Approximately 50% of cancer patients in China have gastrointestinal tumors, mainly in GC, and the 5-year survival rate is less than 35% [2]. GC has a multistep progression [3]. The 5-year survival rate of patients with advanced GC is less than 20%, but it may reach more than 90% if it only invades the mucosal or submucosal layer [4]. However, GC has no specific clinical symptoms in the early stage, and most patients are in the middle and advanced stage when diagnosed, which leads to a poor prognosis. Therefore, it is imperative to explore the mechanism of GC and identify biomarkers for early diagnosis.

Metabolomics is a novel technique that explores the biological states of metabolites in tissue extracts and body fluids such as plasma, serum and urine [5,6] and has been used to characterize metabolic disorders and identify potential biomarkers of various cancers [7–9]. Early studies on GC using metabolomics were mainly performed on plasma [10–12], serum [13,14], and urine [15] samples, and there were few studies involved in GC tissues. Metabolic profiling of patients with GC using tissue samples with and without lymph node metastasis (LNM) was performed, and some differential metabolites were identified as potential factors for the diagnosis and prognosis of GC patients with or without LNM [16]. The present study aimed to screen differential metabolites and reveal metabolic pathways related with GC with the help of transcriptomics data from TCGA.

## Materials and methods

### Patient selection

Patients recruited in the study were diagnosed with GC according to results of a gastroscopy examination and a biopsy. Inclusion criteria included (i) aged between 20 and 80 years old, both male and female; (ii) primary tumor; and (iii) no previous treatment for cancer such as radiation, operation, and chemoradiotherapy. Exclusion criteria included (i) metabolic disease including hyperlipoidemia, diabetes mellitus and gout; (ii) congenital diseases; (iii) severe gastric cancer (survival < 2 months); and (iv) distant metastases.

The present study was approved by the Ethics Committee of Henan Provincial People’s Hospital, and all subjects provided informed consent. The registration number is ChiCTR2100041912.

### Sample collection

GC and matched paracancerous (PC) tissues were collected from dissected specimens of patients undergoing radical gastrectomy of the same anatomical region and they were obtained from mucosa to muscle layers but serosa was removed. PC tissues were 5 cm from the cancer tissues. Samples were washed immediately with phosphate buffered saline and frozen in liquid nitrogen and stored at −80°C. The pathological stages were determined based on the tumor node metastasis (TNM) staging system from the American Joint Cancer Committee/Union for International Cancer Control (AJCC/UICC).

### Preparation and LC-MS/MS analysis of tissue samples

Prior to analysis, 50 mg of the tissue sample was weighed and 1000 μl of extract solution (acetonitrile: methanol: water = 2:2:1, with an isotope-labeled internal standard mixture) was added. After vortexing for 30 s, samples were grind for 4 min and homogenized by sonicating for 5 min in an ice-water bath. These steps were repeated three more times. Samples were incubated for 1 h at −40°C and centrifuged at 12000 rpm for 15 min at 4°C. The supernatant was transferred to a fresh glass vial for analysis.

LC-MS/MS analysis were performed using an ultra-high-performance liquid chromatography (UHPLC) system (Vanquish, Thermo Fisher Scientific) with a UPLC BEH amide column (2.1 mm * 100 mm, 1.7 μm) coupled to the Q Exactive HFX mass spectrometer (Orbitrap MS, Thermo). The mobile phase was composed of 25 mmol/L ammonium acetate and 25 ammonia hydroxide in water (pH 9.75) and acetonitrile [17]. The injection volume was 3 ml and the auto-sampler temperature was 4°C. The Q Exactive HFX mass spectrometer was used to acquire MS/MS spectra in information-dependent acquisition (IDA) mode in the control panel of the acquisition software (Xcalibur, Thermo). In this mode, the acquisition software continuously evaluated the full-scan MS spectrum. The ESI source conditions were set as follows: sheath gas flow rate: 25 Arb, Aux gas flow rate: 20 Arb, capillary temperature: 350°C, full MS resolution: 60000, MS/MS resolution: 7500, collision energy: 10/30/60 in NCE mode, and spray voltage: 3.6 kV (positive) or -3.2 kV (negative).

### Data preprocessing and annotation

The original data were converted to mzXML format by ProteoWizard and R package XCMS (version 3.2) [18]. An in-house program was used for peak detection, extraction, alignment, and integration. Then an in-house MS2 database (BiotreeDB, V2.1) was used for in metabolite annotation [18]. The cutoff for annotation was set at 0.3.

### Statistical analysis

The data were processed using the SIMCA 16.0.2 software package (Sartorius Stedim Data Analytics AB, Umea, Sweden) [19,20] for principal component analysis (PCA) and orthogonal projections to latent structures-discriminant analysis (OPLS-DA). As an unsupervised pattern recognition method, PCA shows the distribution of origin data and general separation. OPLS-DA was used to obtain maximal covariance between variables and the sample category in both positive and negative models. Seven-fold cross-validation and 200 permutation tests were used to estimate the robustness and the predictive ability of our model [21].

Variables with variable importance in the projection (VIP) > 0.5 were defined as key metabolites. MetaboAnalyst (https://www.metaboanalyst.ca/) was used to perform a search for metabolite pathways based on key metabolites, and metabolite pathways that satisfied the condition of *P*<0.05 were chosen for further analysis.

### Integration of metabolomics and transcriptomics data

Genes related to metabolite pathways were identified using the KEGGREST function of the R software package. GC transcriptomic data were obtained from The Cancer Genome Atlas (TCGA) and comprised 375 GC and 32 non-tumor tissues. The DESeq2 R package was used to conduct normalization and differential gene expression analysis, and we specified |log_2_FC| (fold change) > 2 and the *P* value < 0.05 as cutoffs to identify differentially expressed genes (DEGs). A Venn diagram was generated by VENNY 2.1 to obtain overlapping genes.

### Identification of key signaling pathways, genes, and metabolites

String (https://string-db.org/) was used to find Kyoto Encyclopedia of Genes and Genomes (KEGG) pathways indicating the interaction between proteins. KEGG pathways were obtained using String to identify signaling pathways enriched by overlapping genes. The diagram of the metabolic pathway with the minimum value, glycerophospholipid (GPL) metabolism, for the false discovery rate (FDR) was drafted. The protein expression of genes related to GPL metabolism between GC and normal tissues was determined using immunohistochemistry (IHC) from the Human Protein Atlas database (HPA) (https://www.proteinatlas.org/).

## Results

### Baseline clinical characteristics of patients

A total of 28 GC tissues and 28 matched PC tissues were collected. Characteristics of subjects were showed in Table 1, including age, gender, weight, height, body mass index (BMI), hobbies, types of tumor localization, and pathologic tumor stages.

**Table 1:** The characteristics summary of subjects

Characteristics	Patients (*n*=28)
Age (years, means ± SD)	60 ± 9
Gender (female/male)	9/19
Weight (kg, means ± SD)	58.6
Height (m, means ± SD)	1.65
BMI (kg/m^2^, means ± SD)	21.62
Hobbies	
Smoking	15
Drinking	10
Tumor localizations, no	
Cardia	5
Fundus of stomach	1
Body of stomach	5
Lesser curvature	8
Antrum	9
Pathologic tumor stages, no	
I (IA, IB)	1
II (IIA, IIB)	7
III (IIIA, IIIB)	10
IV (IVA, IVB)	10

Note: BMI, body mass index.

### Metabolic profiles of gastric cancer

**Figure 1:**
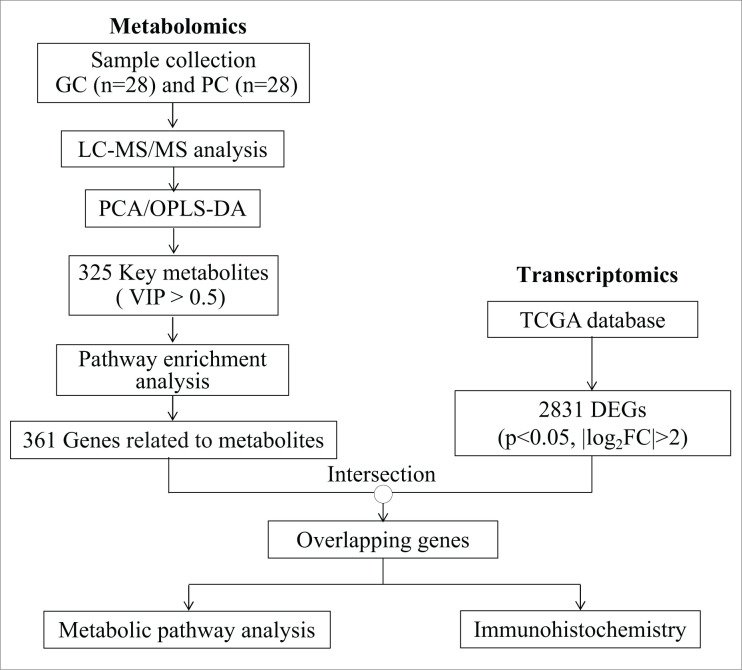
An overview of study profile for identifying metabolites and metabolic pathways of GC

An overview of the study profile is shown in Figure 1. A total of 4893 peaks (negative ion mode: 2163, positive ion mode: 2730) were obtained, and 485 metabolites (negative ion mode: 145, positive ion mode: 340) were identified. There were different mass spectrum peaks identified between the GC and PC groups, as shown in Supplementary Figure S1. Examination of the PCA score plots (Supplementary Figure S2a–d) showed that most of the samples were within a 95% confidence interval (CI) but failed to provide satisfactory separation of data. Permutation tests of the OPLS-DA models for the two groups were carried out to prevent overfit of models (Supplementary Figure S2e and S2f), and the results showed that the models have good predictability and do not overfit.

**Figure 2:**
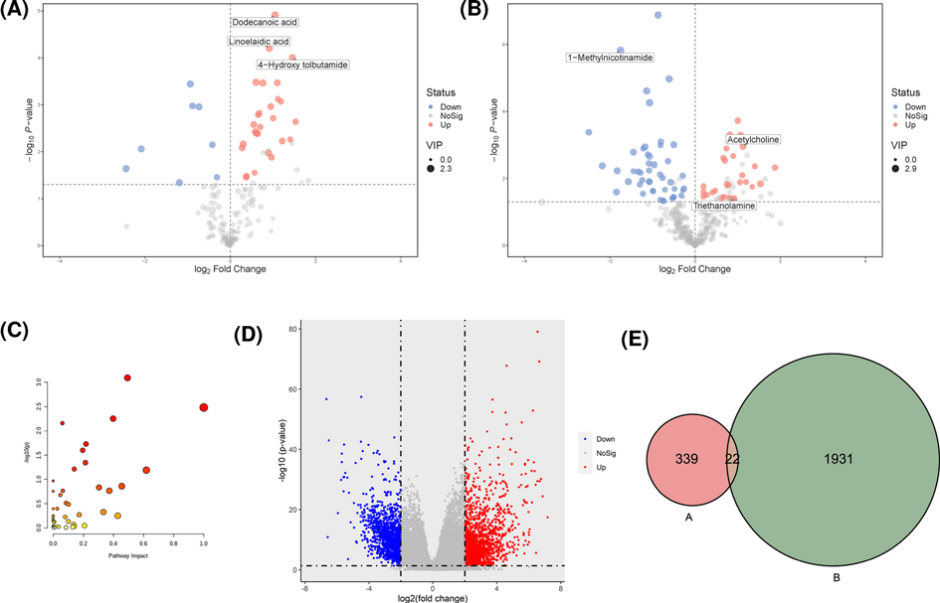
Integration analysis of metabolomics and transcriptomics data (**A**) Volcano plots derived from negative ion mode of GC patients. (**B**) Volcano plot derived from positive ion mode of GC patients. The red and blue points represented up-regulated and down-regulated genes, respectively. (**C**) Bubble analysis of metabolic pathways between GC and PC groups. Ordinate showed the significance and abscissa represented the impact of pathway. (**D**) Volcano plot of DEGs from TCGA. The red and blue points represented up-regulated and down-regulated genes, respectively. (**E**) Venn diagram of metabolic pathways related genes and DEGs. (A) represented genes related with metabolites and (B) indicated DEGs obtained from TCGA.

A total of 325 key metabolites (Supplementary Table S1) were identified under the condition of VIP > 0.5. Of these metabolites, there were 35 significantly different peaks (27 up-regulated and 8 down-regulated) in the negative ion model, and 76 significantly different peaks (29 up-regulated and 47 down-regulated) in the positive ion model (*P*<0.05 and VIP > 1, Figure 2A,B).

### Pathway enrichment analysis

Pathway enrichment analysis was performed to identify signaling pathways related to key metabolites. A total of 48 metabolic pathways were found (Figure 2C and Table 2); of these, seven were chosen (*P*<0.05) for further analysis, including alanine, aspartate and glutamate metabolism, phenylalanine, tyrosine and tryptophan biosynthesis, glycerophospholipid metabolism, pantothenate and CoA biosynthesis, purine metabolism, arginine and proline metabolism and sphingolipid metabolism. The KEGGREST function of the R software package was used to identify genes involved in the seven metabolite pathways, and 361 genes were obtained after removal of duplicates (Supplementary Table S2).

**Table 2:** Details of pathway analyses based on metabolomics data

Pathway description	Total	Count	*P*.value	FDR
Alanine, aspartate, and glutamate metabolism	28	9	0.0008	0.0681
Phenylalanine, tyrosine, and tryptophan biosynthesis	4	3	0.0033	0.1389
Glycerophospholipid metabolism	36	9	0.0056	0.1459
Pantothenate and CoA biosynthesis	19	6	0.0069	0.1459
Purine metabolism	65	12	0.0187	0.3138
Arginine and proline metabolism	38	8	0.0251	0.3521
Sphingolipid metabolism	21	5	0.0451	0.5414
Histidine metabolism	16	4	0.0610	0.5987
Phenylalanine metabolism	10	3	0.0641	0.5987
D-glutamine and D-glutamate metabolism	6	2	0.1077	0.9043
Beta-alanine metabolism	21	4	0.1380	0.9817
Arginine biosynthesis	14	3	0.1466	0.9817
Butanoate metabolism	15	3	0.1710	0.9817
Nicotinate and nicotinamide metabolism	15	3	0.1710	0.9817
Aminoacyl-tRNA biosynthesis	48	7	0.1753	0.9817
Cysteine and methionine metabolism	33	5	0.2088	1.0000
Ether lipid metabolism	20	3	0.3044	1.0000
Pyrimidine metabolism	39	5	0.3238	1.0000
Glycine, serine, and threonine metabolism	33	4	0.3984	1.0000
Linoleic acid metabolism	5	1	0.3993	1.0000
Arachidonic acid metabolism	36	4	0.4662	1.0000
Pentose and glucuronate interconversions	18	2	0.5324	1.0000
Ascorbate and aldarate metabolism	8	1	0.5579	1.0000
Valine, leucine and isoleucine biosynthesis	8	1	0.5579	1.0000
Taurine and hypotaurine metabolism	8	1	0.5579	1.0000
Citrate cycle (TCA cycle)	20	2	0.5912	1.0000
Ubiquinone and other terpenoid-quinone biosynthesis	9	1	0.6009	1.0000
Propanoate metabolism	23	2	0.6688	1.0000
Biosynthesis of unsaturated fatty acids	36	3	0.6934	1.0000
Glycolysis/Gluconeogenesis	26	2	0.7341	1.0000
Alpha-linolenic acid metabolism	13	1	0.7352	1.0000
Galactose metabolism	27	2	0.7533	1.0000
Glycosylphosphatidylinositol (GPI)-anchor biosynthesis	14	1	0.7610	1.0000
Starch and sucrose metabolism	18	1	0.8416	1.0000
Pentose phosphate pathway	22	1	0.8952	1.0000
Pyruvate metabolism	22	1	0.8952	1.0000
Fatty acid degradation	39	2	0.9051	1.0000
Tyrosine metabolism	42	2	0.9262	1.0000
Folate biosynthesis	27	1	0.9375	1.0000
Phosphatidylinositol signaling system	28	1	0.9437	1.0000
Fatty acid biosynthesis	47	2	0.9519	1.0000
Inositol phosphate metabolism	30	1	0.9542	1.0000
Glyoxylate and dicarboxylate metabolism	32	1	0.9628	1.0000
Amino sugar and nucleotide sugar metabolism	37	1	0.9779	1.0000
Fatty acid elongation	39	1	0.9821	1.0000
Valine, leucine, and isoleucine degradation	40	1	0.9839	1.0000
Primary bile acid biosynthesis	46	1	0.9914	1.0000
Steroid hormone biosynthesis	85	1	0.9999	1.0000

Note: FDR, false discovery rate.

### Differentially expressed genes in GC

To explore genes related to the metabolism of GC and establish relationships between genes and metabolites, DEGs between GC and non-tumor tissues from the TCGA were screened. A total of 2831 DEGs were identified as shown in Figure 2D, of which 1195 were down-regulated and 1636 were up-regulated. 878 DEGs were failed to be identified as gene, then excluded. So, 1953 DEGs were included in further analysis.

### Potential biomarker analysis

**Figure 3:**
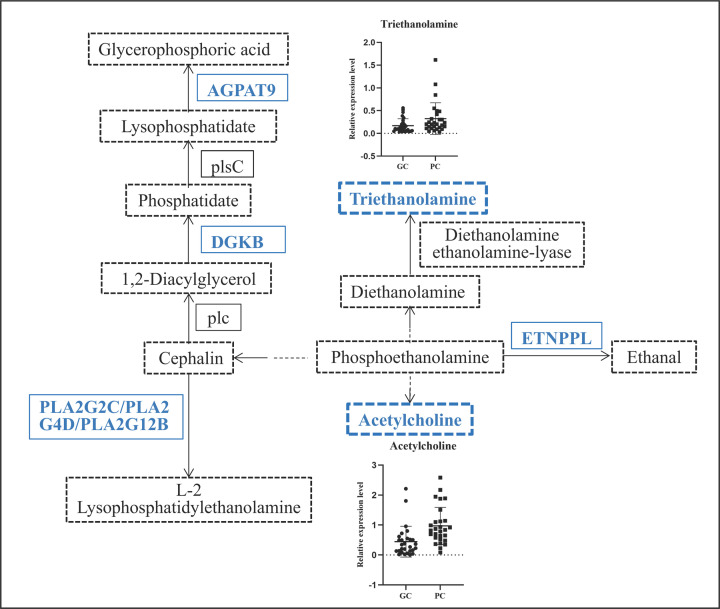
Regulatory pathway diagram of glycerophospholipid metabolism The blue words represent down-regulation of genes or metabolites in GC.

**Figure 4:**
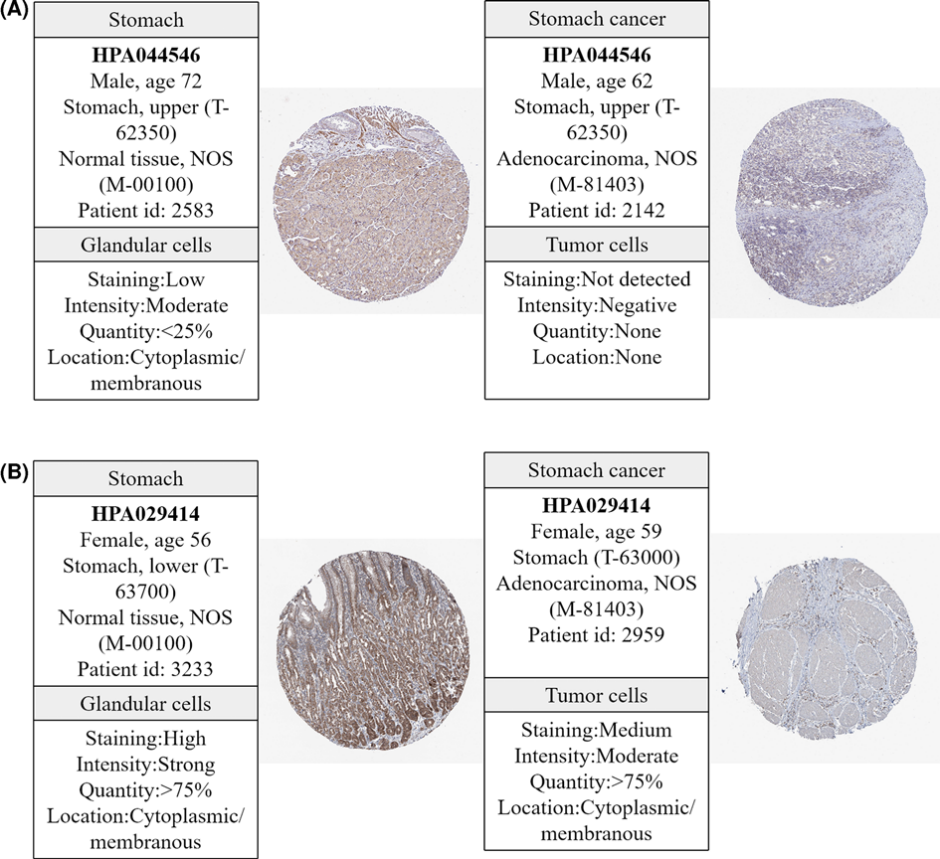
Immunohistochemistry of genes related to glycerophospholipid metabolism from the HPA database (**A**) ETNPPL in GC (right) and normal tissues (left); (**B**) AGPAT9 in GC (right) and normal tissues (left).

To explore the relationship between metabolites and genes, we looked for overlap between genes related to metabolites and DEGs obtained from the TCGA cohort and found 22 gene were overlapping genes (Figure 2E and Table 3). String was used to explore the interaction networks of 22 overlapping genes, and 23 pathways were enriched in total (Table 4). GPL metabolism with the minimum FDR value contains six genes (PLA2G2C, PLA2G4D, PLA2G12B, ETNPPL, DGKB, and AGPAT9) and two metabolites (acetylcholine and triethanolamine) and is considered a pathway that affects the progression of GC. The six genes and two metabolites in GPL metabolism were down-regulated (Figure 3). Moreover, the IHC staining obtained from the HPA database showed lower expression of ETNPPL and AGPAT9 in GC tissue than in normal tissues (Figure 4).

**Table 3:** Overlapped genes among metabolic pathways related genes and DEGs

Genes	logFC	*P*.value	Genes	logFC	*P*.value
CERS3	−3.5985	6.90E-08	ENPP3	−3.1016	1.55E-40
ASAH2	−4.2801	4.32E-62	GPAT3	−2.8780	8.69E-22
ACER1	−6.3393	3.82E-20	DGKB	−3.2171	1.49E-15
ENPP7	−3.1016	9.60E-59	PLA2G12B	−2.0154	4.38E-06
PSAPL1	2.0386	4.23E-11	PLA2G2C	−2.0147	1.31E-15
CKM	−3.5381	7.80E-27	PLA2G4D	−2.0143	5.44E-10
CKMT2	−3.5353	2.49E-25	ETNPPL	−3.0742	1.54E-10
CKB	−3.5423	1.22E-17	PAH	−2.0414	5.25E-11
NOS1	−2.0788	6.66E-13	GPT	−2.8628	1.87E-12
NT5C1A	−2.0720	8.39E-15	RIMKLB	2.0639	2.57E-19
AMPD1	−4.8170	3.96E-17	ASPA	−4.2283	2.50E-16

Note: FC, fold change.

**Table 4:** Signaling pathways focused by overlapped genes

ID	Pathway description	Observed gene count	FDR	Matching proteins
hsa01100	Metabolic pathways	21	1.08E-22	CKM, NT5C1A, PLA2G2C, ASPA, CERS3, PLA2G4D, ETNPPL, CKB, ACER1, ENPP7, RIMKLB, PLA2G12B, GPT, ASAH2, DGKB, CKMT2, ENPP3, AMPD1, PAH, NOS1, AGPAT9
hsa00564	Glycerophospholipid metabolism	6	3.23E-08	PLA2G2C, PLA2G4D, ETNPPL, PLA2G12B, DGKB, AGPAT9
hsa00330	Arginine and proline metabolism	4	4.40E-06	CKM, CKB, CKMT2, NOS1
hsa00600	Sphingolipid metabolism	4	4.40E-06	CERS3, ACER1, ENPP7, ASAH2
hsa00592	Alpha-linolenic acid metabolism	3	4.27E-05	PLA2G2C, PLA2G4D, PLA2G12B
hsa00591	Linoleic acid metabolism	3	5.37E-05	PLA2G2C, PLA2G4D, PLA2G12B
hsa00250	Alanine, aspartate, and glutamate metabolism	3	7.80E-05	ASPA, RIMKLB, GPT
hsa00565	Ether lipid metabolism	3	0.00015	PLA2G2C, PLA2G4D, PLA2G12B
hsa00590	Arachidonic acid metabolism	3	0.00029	PLA2G2C, PLA2G4D, PLA2G12B
hsa00220	Arginine biosynthesis	2	0.0015	GPT, NOS1
hsa04071	Sphingolipid signaling pathway	3	0.0015	CERS3, ACER1, ASAH2
hsa04270	Vascular smooth muscle contraction	3	0.0015	PLA2G2C, PLA2G4D, PLA2G12B
hsa00760	Nicotinate and nicotinamide metabolism	2	0.0024	NT5C1A, ENPP3
hsa00230	Purine metabolism	3	0.0037	NT5C1A, ENPP3, AMPD1
hsa04975	Fat digestion and absorption	2	0.0037	PLA2G2C, PLA2G12B
hsa00561	Glycerolipid metabolism	2	0.0071	DGKB, AGPAT9
hsa04014	Ras signaling pathway	3	0.0071	PLA2G2C, PLA2G4D, PLA2G12B
hsa04730	Long-term depression	2	0.0071	PLA2G4D, NOS1
hsa01230	Biosynthesis of amino acids	2	0.0088	GPT, PAH
hsa04972	Pancreatic secretion	2	0.0142	PLA2G2C, PLA2G12B
hsa00240	Pyrimidine metabolism	2	0.0143	NT5C1A, ENPP3
hsa05231	Choline metabolism in cancer	2	0.0143	PLA2G4D, DGKB
hsa04072	Phospholipase D signaling pathway	2	0.0275	PLA2G4D, DGKB

Note: FDR, false discovery rate.

## Discussion

In the present study, we performed metabolic profiling of GC using GC and PC tissues and found that certain metabolites were significantly different. Twenty-two genes related to metabolites were identified based on metabolomics analysis with the help of TCGA transcriptomics data. GPL metabolism, which involves in six genes (PLA2G2C, PLA2G4D, PLA2G12B, ETNPPL, DGKB, and AGPAT9) and two metabolites (acetylcholine and triethanolamine), is likely associated with GC. The protein expression of AGPAT9 and ETNPPL were decreased in GC tissues.

The occurrence and development of cancers are dependent on molecular alterations and multiple of omics technologies, including metabolomics, transcriptomics, genomics and proteomics, have been performed to elucidate the mechanisms of cancer [22]. Transcriptomics analysis of cancers provides an orthogonal perspective for the expression of metabolic genes to investigate metabolism [23]. Some studies combined results of single-level omics with bioinformatics data available from websites, such as the TCGA and GEO, to screen biomarkers or explore the pathogenesis of cancer. The use of a combination of metabolomics and transcriptomics data from the TCGA or GEO is relatively frequent. Purine metabolism and related genes AMPD1 and RRM2 were enriched by combining metabolomics and transcriptomics data from the TCGA and GEO in breast cancer [24]. Differential metabolites in non-small cell lung cancer (NSCLC) were identified that were further verified by transcriptomics analysis of TCGA data, and a fully connected network of metabolites and genes in NSCLC was generated [25]. Sphingolipids were identified in breast cancer patients, and genes related to metabolism, such as CERK, SPHK1, and SGMS1, were verified by a TCGA cohort [26]. We performed metabolic profiling of GC and several metabolic pathways, and related genes and metabolites were identified with the help of TCGA transcriptomics data in our study.

Metabolomics research is committed to identifying and developing metabolically active targets in cancer therapeutics and pharmacology [27]. Many studies have shown the tremendous potential of metabolomics in GC. The urinary metabolomic profile of GC was explored and 77 metabolites were identified. A parsimonious biomarker profile of GC was investigated, and model performance was assessed [15]. Serum metabolites in patients with GC and their relationship with the prognosis of GC were investigated. Researchers found that three metabolites, 2,4-hexadienoic acid, 4-methylphenyl dodecanoate, and glycerol tributanoate, were related to the prognosis of GC [28]. Differential metabolites and metabolic pathways were identified in GC and its adjacent tissues in our study. GPL metabolism was enriched, and two metabolites involved in GPL metabolism, acetylcholine and triethanolamine, were differently expressed between the GC and PC groups. Because GPL is the most abundant lipid, its metabolism is closely related to oncogenesis and progression since the purpose of abnormal lipid metabolism is to synthesize more cell membranes lipids to meet the needs of the rapid proliferation of cancer cells and their increased demand for energy [29,30]. GPL metabolism has been reported to be dysregulated in many cancers, including NSCLC [31], melanoma [32], glioma [33], prostate cancer [34], colorectal cancer [35], and oral squamous cell carcinoma [36]. Therefore, GPL metabolism is likely associated with GC.

As one of the hallmarks of cancer [37], altered metabolism is regulated by genetic alterations to meet increased energy demands [38]. Changes in key genes had an impact on metabolic pathways to some extent [39]. A previous study showed that fatty acid metabolism was enriched by PHTF2 in GC and lipid metabolism regulated by PHTF2 significantly affected the tumorigenesis of GC cells [40]. Our study found that six genes (PLA2G2C, PLA2G4D, PLA2G12B, ETNPPL, DGKB, and AGPAT9) were altered in the GPL metabolic pathway. Researchers suggested that AGPAT9 may be correlated with cancer risk [41,42]. AGPAT9 is considered a hub gene that may exhibit significant prognostic potential for clear cell renal cell carcinoma because of its relation to immune infiltration [43]. A study found that AGPAT9 inhibited proliferation, migration, and invasion of breast cancer cell indicated that increasing AGPAT9 expression may be a new approach for breast cancer treatment [44]. ETNPPL is underexpressed in primary glioblastoma (GBM) and shows potential diagnostic implication for GBM [45]. Overexpression of ETNPPL reduced the growth of glioma stem cells and ETNPPL expression was inversely correlated to glioma grade [46]. Summarily, AGPAT9 and ETNPPL are anti-oncogenes of cancer. Our study showed that AGPAT9 and ETNPPL were down-regulated in patients with GC and participated in the regulation of GPL metabolism, and the protein expression of AGPAT9 and ETNPPL was lower in GC than in normal tissues, which is consistent with other studies.

There are some limitations of our study. First, the sample size was relatively small, although the GC and PC tissues were matched. Second, all patients were recruited from a single center, which might limit their generalization. Third, GC is very heterogenous due to anatomic location, molecular classification, and etiologies, but we failed to group those factors when identifying metabolites. Stratified analysis based on anatomic location, molecular classification, and etiologies is worth expecting to identify metabolites and metabolic pathways of GC. Our study may be regarded as a preliminary study exploring metabolic characteristics of GC and PC tissues, and further research with a larger sample, multiple research centers, and verification using other omics technologies is necessary to confirm our results. In conclusion, we investigated the tissue-based metabolomics profile of GC, and several differential metabolites were identified. GPL metabolism may affect the progression of GC with the help of TCGA transcriptomics data.

## Supplementary Material

Supplementary Figures S1-S2 and Tables S1-S2

## Data Availability

Metabonomics data is available in the open-access repository MetaboLights, study ID: MTBLS3303.

## Abbreviations

BMI: body mass index
CI: confidence interval
DEG: differentially expressed gene
FC: fold change
FDR: false discovery rate
GBM: glioblastoma
GC: gastric cancer
GPI: glycosylphosphatidylinositol
GPL: glycerophospholipid
NSCLC: non-small cell lung cancer
PC: paracancerous
PCA: principal component analysis
TCGA: The Cancer Genome Atlas
TNM: tumor node metastasis
