# Supplementary material for: Tissue-based metabolomics reveals metabolic signatures and major metabolic pathways of gastric cancer with help of transcriptomic data from TCGA
Source: Biosci Rep. 2021 Oct 4;41(10):BSR20211476. doi: 10.1042/BSR20211476 (PMC8490861; doi:10.1042/BSR20211476)
Supplement: Supplementary Figures S1-S2 and Tables S1-S2 [file BSR-2021-1476_supp.pdf]

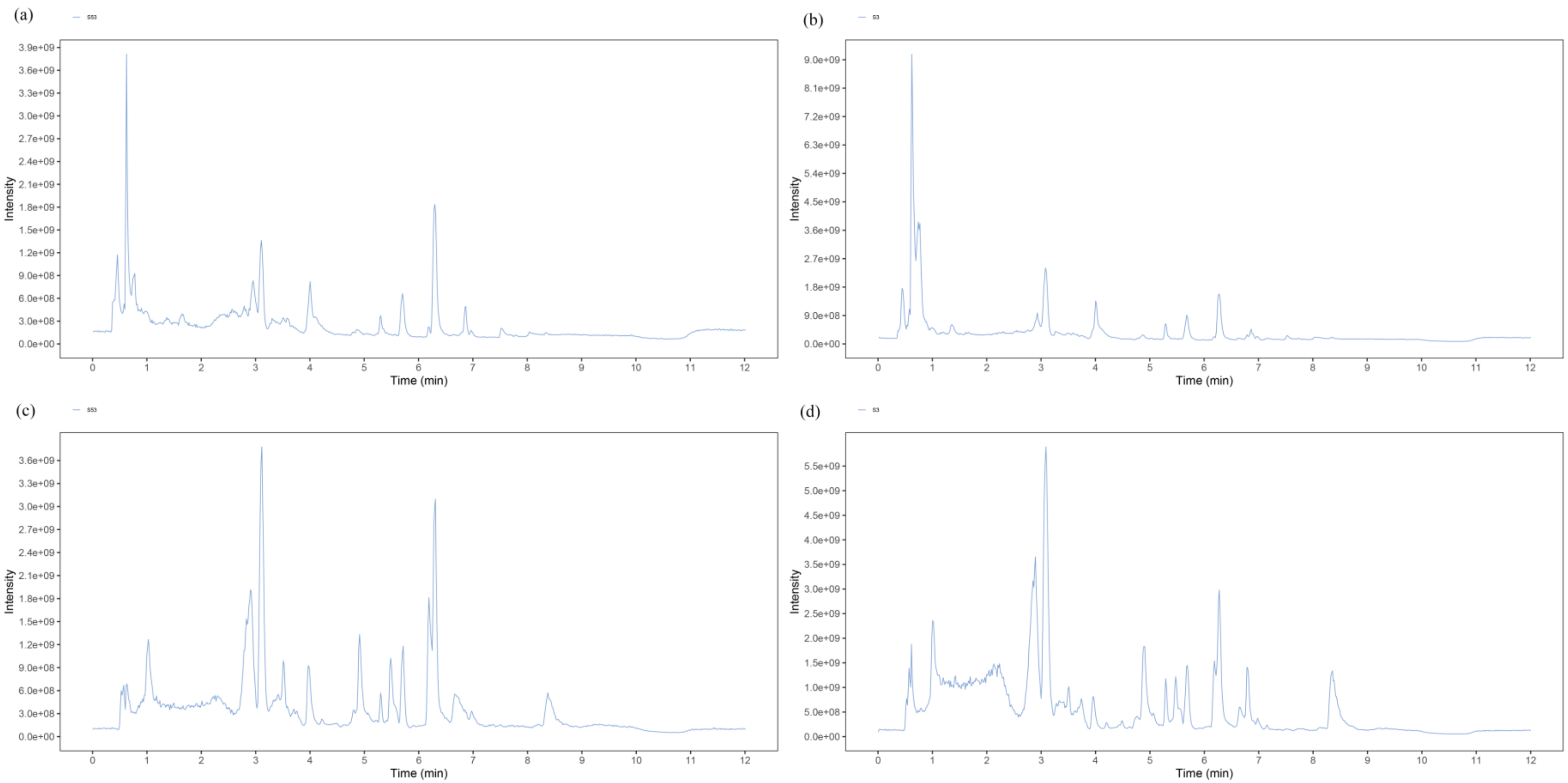

Fig S1 UHPLC-QE-MS ion chromatograms of GC and PC tissue. Negative ion mode of GC tissue (a) and PC tissue (b), and positive ion mode of GC tissue (c) and PC tissue (d) of the same patient.

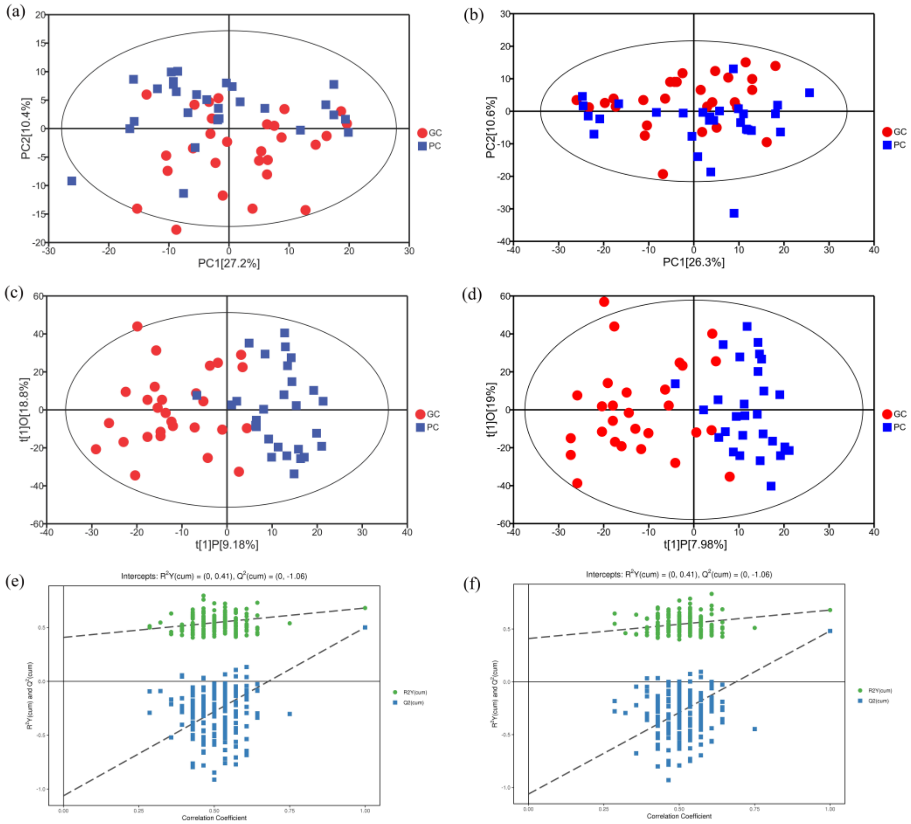

Fig S2 Identification of discriminating biomarkers by metabolome analysis. The PCA (a) and OPLS-DA (c) score plot and OPLS-DA (e) model are in negative ion mode. PCA (b) and OPLS-DA (d) score plot and OPLS-DA (f) model are in positive ion mode. The abscissa represents correlation between random group and original group, and ordinate represents scores of R2Y and Q2. When permutation retention is 1, the values of Q2 and R2Y are close to 0.5 and 1 respectively, and Q2 is with positive slope showing that models have good predictability and do not overfit.

Table S1 Details of 325 key metabolites identified by metabonomics

Ionization models	Metabolites	rt	m/z	Relative expression		VIP	P value	LogFC
				GC group	PC group			
NEG	Dodecanoic acid	49.80	199.17	2.91	1.40	2.31	0.00001	1.05
NEG	Linoelaidic acid	39.75	279.23	136.28	72.19	2.03	0.00006	0.92
NEG	4-Hydroxy tolbutamide	278.53	285.09	0.96	0.35	1.86	0.00010	1.46
NEG	3-Hydroxymethylglutaric acid	412.25	161.04	1.61	1.06	2.28	0.00033	0.60
NEG	Ethyl dodecanoate	39.76	227.20	3.98	1.85	1.78	0.00034	1.10
NEG	myo-Inositol	412.20	179.06	19.74	11.63	2.29	0.00034	0.76
NEG	Deoxyinosine	199.11	251.08	0.17	0.33	2.17	0.00036	-0.94
NEG	Alpha-dimorphecolic acid	55.35	295.23	1.75	0.81	1.22	0.00076	1.12
NEG	5Z-Dodecenoic acid	50.21	197.15	0.76	0.33	1.39	0.00085	1.19
NEG	N-Acetyl-glucosamine 1-phosphate	466.09	300.05	0.15	0.28	1.71	0.00106	-0.88
NEG	Isopalmitic acid	39.75	255.23	113.86	58.91	1.77	0.00109	0.95
NEG	m-Coumaric acid	61.15	163.04	0.70	1.16	2.04	0.00111	-0.73
NEG	D-Xylitol	101.23	151.06	2.47	1.54	1.46	0.00151	0.68
NEG	Prostaglandin E2	47.35	351.22	0.47	0.30	1.32	0.00164	0.66
NEG	Palmitoleic acid	40.67	253.22	16.46	8.19	1.47	0.00193	1.01
NEG	beta-D-Glucosamine	415.01	214.05	3.61	1.24	1.41	0.00230	1.54
NEG	Oleic acid	100.03	281.25	2.56	1.74	1.84	0.00264	0.55
NEG	Ricinoleic acid	55.24	297.24	0.50	0.31	1.34	0.00298	0.70
NEG	Bovinic acid	205.20	279.23	0.65	0.43	1.79	0.00374	0.59
NEG	2-Keto-3-deoxy-D-gluconic acid	152.33	177.04	3.24	2.12	1.98	0.00404	0.61
NEG	N-Acetylaspartylglutamic acid	463.91	303.08	0.14	0.09	1.92	0.00413	0.64
NEG	Prostaglandin D2	75.64	351.22	0.52	0.19	1.15	0.00551	1.41
NEG	Resolvin D2	45.48	375.22	0.64	0.27	1.78	0.00598	1.22

NEG	D-Glutamine	411.50	145.06	3.31	2.68	2.06	0.00692	0.31
NEG	16-Hydroxy hexadecanoic acid	57.48	271.23	0.65	0.86	1.60	0.00715	-0.42
NEG	Uridine	177.06	243.06	40.32	33.16	1.89	0.00831	0.28
NEG	Cholesterol sulfate	25.96	465.30	6.51	27.67	2.02	0.00876	-2.09
NEG	Guanine	253.11	150.04	0.56	0.30	1.91	0.01065	0.90
NEG	Cytidine	262.43	242.08	0.08	0.04	1.77	0.01316	0.97
NEG	Ethyl glucuronide	65.42	221.07	0.05	0.28	2.08	0.02295	-2.44
NEG	N-Acetyl-L-aspartic acid	414.61	174.04	3.76	2.53	1.13	0.02818	0.57
NEG	Hypogeic acid	99.77	253.22	0.51	0.39	1.46	0.03328	0.38
NEG	Beta-Guanidinopropionic acid	372.07	130.06	3.32	2.56	1.72	0.03510	0.37
NEG	Gluconolactone	50.28	177.04	0.88	1.09	1.36	0.03517	-0.31
NEG	Imidazoleacetic acid	71.72	125.03	0.59	1.34	2.01	0.04592	-1.20
POS	PC(16:0/16:0)	176.63	734.57	4.87	8.89	2.86	0.00000	-0.87
POS	1-Methylnicotinamide	324.25	137.07	2.29	7.68	2.89	0.00000	-1.75
POS	PC(20:1(11Z)/20:4(5Z,8Z,11Z,14Z))	166.83	836.61	0.70	1.07	2.79	0.00001	-0.61
POS	PC(24:1(15Z)/18:4(6Z,9Z,12Z,15Z))	165.04	864.64	0.03	0.06	2.71	0.00002	-1.13
POS	PC(15:0/15:0)	177.98	706.54	0.94	1.96	2.73	0.00006	-1.07
POS	gamma-Glutamylleucine	436.56	261.14	0.05	0.02	1.78	0.00019	1.00
POS	PC(P-18:1(11Z)/22:2(13Z,16Z))	166.81	824.65	0.02	0.10	1.98	0.00042	-2.49
POS	N4-Acetylcytidine	344.12	286.10	0.49	0.28	1.67	0.00050	0.82
POS	Camelinin	421.21	262.13	0.32	0.15	2.17	0.00051	1.07
POS	PC(20:3(8Z,11Z,14Z)/20:1(11Z))	39.64	838.63	0.16	0.28	1.99	0.00081	-0.80
POS	PC(20:5(5Z,8Z,11Z,14Z,17Z)/20:1(11Z))	166.81	834.60	0.93	1.31	2.32	0.00099	-0.50
POS	Carvyl propionate	34.12	209.15	0.01	0.02	1.29	0.00103	-0.81
POS	Acetylcholine	226.98	146.12	0.97	0.45	2.21	0.00111	1.12
POS	PC(16:0/15:0)	177.54	720.55	0.22	0.51	2.49	0.00115	-1.17
POS	PE(22:4(7Z,10Z,13Z,16Z)/14:0)	173.08	740.52	1.08	0.65	1.31	0.00128	0.74
POS	Dehydrophytosphingosine	154.25	316.28	0.01	0.02	1.32	0.00128	-1.22

POS	PC(14:0/14:0)	179.35	678.50	0.03	0.08	2.25	0.00164	-1.44
POS	5'-Methylthioadenosine	93.99	298.10	0.11	0.23	1.53	0.00192	-1.11
POS	PC(20:5(5Z,8Z,11Z,14Z,17Z)/15:0)	171.28	766.54	0.98	0.53	1.11	0.00212	0.90
POS	PC(22:6(4Z,7Z,10Z,13Z,16Z,19Z)/22:2(13Z,16Z))	163.23	886.63	0.02	0.05	2.33	0.00219	-1.07
POS	p-Cresol glucuronide	217.42	285.10	0.05	0.03	1.53	0.00246	0.67
POS	Adenine	231.63	136.06	1.90	1.17	1.71	0.00292	0.70
POS	PC(18:2(9Z,12Z)/P-18:1(11Z))	167.72	768.59	0.91	1.41	1.73	0.00306	-0.63
POS	PE(P-18:1(9Z)/16:1(9Z))	172.19	700.53	0.17	0.37	1.76	0.00354	-1.08
POS	PC(18:1(11Z)/14:0)	175.74	732.55	3.39	6.60	2.39	0.00389	-0.96
POS	Sphinganine	134.34	302.30	0.04	0.17	2.45	0.00419	-2.18
POS	Iminoerythrose 4-phosphate	434.61	200.03	0.15	0.06	1.37	0.00433	1.41
POS	8-Acetoxy-4'-methoxypinoresinol 4-glucoside	435.22	593.21	0.02	0.01	1.64	0.00473	1.88
POS	Prolylhydroxyproline	443.81	229.12	0.15	0.32	1.71	0.00578	-1.08
POS	PC(16:0/P-16:0)	173.03	718.57	0.12	0.42	2.66	0.00592	-1.82
POS	Serylalanine	78.45	177.09	0.09	0.23	1.45	0.00610	-1.44
POS	Sphingosine	86.34	300.29	0.68	1.72	2.11	0.00633	-1.33
POS	D-Alanyl-D-alanine	63.32	161.09	0.20	0.47	1.50	0.00681	-1.28
POS	PC(18:1(11Z)/P-16:0)	171.26	744.59	0.60	1.06	1.89	0.00765	-0.82
POS	7-Ketodeoxycholic acid	35.22	424.36	0.10	0.05	1.51	0.00809	1.11
POS	PC(20:4(8Z,11Z,14Z,17Z)/P-18:0)	165.94	794.61	1.72	2.10	1.70	0.00839	-0.29
POS	PC(22:5(4Z,7Z,10Z,13Z,16Z)/20:5(5Z,8Z,11Z,14Z,17Z))	163.18	854.57	0.02	0.04	1.66	0.00963	-0.76
POS	Alanyl-Leucine	43.40	203.14	0.12	0.27	1.66	0.01134	-1.24
POS	2-Pyrrolidinone	394.91	86.06	0.14	0.30	1.84	0.01238	-1.08
POS	N-Acetylputrescine	353.96	131.12	0.05	0.14	2.44	0.01253	-1.56
POS	gamma-Aminobutyric acid	394.91	104.07	0.60	1.25	1.80	0.01271	-1.07
POS	Glycerophosphocholine	451.99	258.11	0.32	0.14	1.23	0.01283	1.20
POS	Carnosine	442.81	227.11	0.06	0.03	1.45	0.01308	1.06
POS	PC(18:0/P-16:0)	172.46	746.60	0.69	1.03	2.11	0.01312	-0.57

POS	NAD	458.77	664.11	0.04	0.01	2.39	0.01448	1.54
POS	L-Gulose	412.12	203.05	0.44	0.26	1.61	0.01470	0.75
POS	Lactosylceramide (d18:1/16:0)	212.84	862.62	0.01	0.04	1.44	0.01534	-1.30
POS	PC(20:2(11Z,14Z)/14:0)	105.24	758.57	21.15	12.30	1.20	0.01626	0.78
POS	L-Acetylcarnitine	329.04	204.12	49.59	43.15	1.49	0.01716	0.20
POS	Koeniginequinone A	429.22	242.08	1.52	0.60	1.10	0.01787	1.34
POS	4-Methoxybenzyl propanoate	409.37	195.10	0.12	0.14	1.64	0.02017	-0.26
POS	PS(18:0/20:4(5Z,8Z,11Z,14Z))	221.68	812.54	0.26	0.36	1.22	0.02060	-0.48
POS	SM(d18:0/14:0)	208.09	677.55	0.04	0.07	1.17	0.02173	-0.70
POS	PI(18:1(11Z)/20:4(5Z,8Z,11Z,14Z))	215.80	885.55	0.04	0.05	1.03	0.02257	-0.28
POS	Pantothenic acid	290.45	220.12	0.15	0.11	1.18	0.02319	0.47
POS	Prolyl-Valine	309.74	215.14	0.03	0.05	1.30	0.02344	-0.99
POS	Valyl-Threonine	57.71	219.13	0.04	0.07	1.33	0.02426	-0.98
POS	4-Hydroxy-2-butenoic acid gamma-lactone	329.04	85.03	0.89	0.78	1.44	0.02491	0.20
POS	PC(16:1(9Z)/14:0)	177.53	704.52	0.07	0.24	2.27	0.02561	-1.84
POS	Succinic acid semialdehyde	375.45	125.02	0.04	0.03	1.21	0.02633	0.41
POS	3,4-Dihydroxy-2-hydroxymethyl-1-pyrrolidinepropanamide	69.43	205.12	0.02	0.06	1.45	0.02872	-1.22
POS	D-erythro-D-galacto-octitol	436.96	243.11	0.02	0.02	1.26	0.03099	0.28
POS	SM(d18:1/14:0)	211.29	675.54	1.07	1.34	1.21	0.03238	-0.32
POS	PE(P-18:1(9Z)/20:4(5Z,8Z,11Z,14Z))	165.05	750.54	4.01	3.49	1.83	0.03390	0.20
POS	Linoelaidyl carnitine	204.48	424.34	3.51	2.19	1.18	0.03511	0.68
POS	N-Methylnicotinamide	46.84	137.07	0.24	0.34	1.45	0.03527	-0.50
POS	Cytarabine	261.97	244.09	0.13	0.08	1.34	0.03715	0.79
POS	PC(18:1(9Z)/P-18:1(11Z))	62.25	770.60	0.27	0.39	1.46	0.03768	-0.51
POS	Glucosyl (2E,6E,10x)-10,11-dihydroxy-2,6-farnesadienoate	485.73	433.24	0.01	0.01	1.45	0.03841	0.63
POS	Dihydrobiopterin	545.04	240.11	0.02	0.01	1.01	0.03948	0.85
POS	PC(20:5(5Z,8Z,11Z,14Z,17Z)/P-18:1(11Z))	164.14	790.57	0.18	0.26	1.36	0.03951	-0.53
POS	Triethanolamine	153.27	150.11	0.32	0.17	1.50	0.03971	0.93

POS	Formiminoglutamic acid	234.48	175.07	0.09	0.15	1.45	0.04346	-0.81
POS	Phosphoribosyl-AMP	444.18	560.08	0.01	0.00	1.32	0.04541	0.92
POS	PE(14:1(9Z)/18:0)	178.74	690.51	0.04	0.07	1.73	0.04631	-0.74
POS	9-O-Methylcoumestrol	355.05	283.06	0.04	0.06	1.34	0.04715	-0.74
NEG	Myristoleic acid	99.98	225.19	0.39	0.35	0.96	0.22221	0.14
NEG	Prostaglandin B2	75.84	333.21	0.42	0.15	0.95	0.00660	1.45
NEG	Undecanoic acid	49.03	185.15	0.49	0.62	0.95	0.05168	-0.34
NEG	Glucose 1-phosphate	498.95	259.02	0.11	0.17	0.92	0.05553	-0.62
NEG	L-Tyrosine	331.08	180.07	0.91	0.95	0.91	0.91608	-0.05
NEG	Inosinic acid	468.37	347.04	0.16	0.05	0.91	0.02455	1.57
NEG	D-Aspartic acid	478.12	132.03	0.31	0.22	0.90	0.08038	0.47
NEG	2-Hydroxystearic acid	55.69	299.26	0.22	0.23	0.89	0.79510	-0.05
NEG	Phenylpyruvic acid	81.88	163.04	0.74	0.99	0.88	0.05802	-0.42
NEG	Pentadecanoic acid	46.84	241.22	1.03	0.74	0.87	0.06250	0.48
NEG	Pelargonic acid	54.31	157.12	15.82	15.26	0.86	0.48368	0.05
NEG	ADP	480.82	426.02	0.06	0.06	0.86	0.99530	0.00
NEG	16(17)-EpDPE	51.67	343.23	0.03	0.04	0.86	0.33807	-0.49
NEG	7-Methylguanosine	218.40	296.10	0.03	0.04	0.86	0.81908	-0.18
NEG	LysoPE(18:1(9Z)/0:0)	228.21	478.29	7.83	2.20	0.83	0.04203	1.83
NEG	D-Malic acid	418.23	133.01	0.09	0.10	0.82	0.33959	-0.16
NEG	Fexofenadine	220.00	500.28	0.56	0.40	0.81	0.61878	0.47
NEG	Pyrrolidonecarboxylic acid	197.04	128.03	0.81	0.78	0.80	0.74960	0.05
NEG	2'-Deoxyguanosine 5'-monophosphate	450.82	346.06	0.40	0.24	0.79	0.14974	0.72
NEG	N-Acetyl-L-alanine	271.49	130.05	0.46	0.63	0.77	0.12301	-0.44
NEG	9-Decenoic acid	54.45	169.12	1.01	0.71	0.77	0.11074	0.50
NEG	Pseudouridine	264.75	243.06	0.25	0.24	0.76	0.84482	0.06
NEG	Succinic acid	408.07	117.02	11.93	11.35	0.76	0.51276	0.07
NEG	2-Hydroxyethanesulfonate	159.35	124.99	6.15	4.85	0.76	0.06300	0.35

NEG	LysoPA(16:0/0:0)	225.45	409.24	0.03	0.02	0.75	0.00942	0.90
NEG	Arachidonic acid	39.03	303.23	103.33	71.26	0.75	0.04725	0.54
NEG	2-Furoic acid	73.05	111.01	0.26	0.26	0.73	0.92862	-0.01
NEG	2-Hydroxy-3-methylbutyric acid	177.41	117.05	2.32	2.63	0.72	0.46134	-0.18
NEG	Hydrocinnamic acid	92.54	149.06	3.43	3.58	0.69	0.97015	-0.06
NEG	Theaflavin	451.34	563.12	0.14	0.14	0.67	0.86140	-0.03
NEG	Capric acid	51.70	171.14	3.95	2.75	0.67	0.13768	0.52
NEG	L-Aspartic acid	432.74	132.03	3.80	2.32	0.67	0.05704	0.71
NEG	Docosahexaenoic acid	39.25	327.23	5.79	5.45	0.65	0.75580	0.09
NEG	Pantothenic acid	292.55	218.10	1.09	0.93	0.65	0.32014	0.23
NEG	6-Keto-prostaglandin F1a	229.39	369.23	0.04	0.05	0.64	0.89050	-0.07
NEG	N-Acetyl-L-methionine	220.88	190.05	0.99	1.00	0.64	0.95089	-0.01
NEG	Isohyodeoxycholic acid	162.59	391.29	0.02	0.13	0.63	0.39206	-2.43
NEG	Prostaglandin D1	110.05	353.23	0.22	0.10	0.63	0.06020	1.12
NEG	Hydrogen phosphate	181.29	96.97	0.20	0.18	0.62	0.20308	0.12
NEG	Hydroxyisocaproic acid	155.89	131.07	1.40	1.90	0.61	0.60818	-0.43
NEG	Tridecanoic acid	37.68	213.19	0.51	0.61	0.60	0.07188	-0.27
NEG	Eicosadienoic acid	38.94	307.26	4.34	3.38	0.59	0.06517	0.36
NEG	L-Cystine	124.20	239.02	0.06	0.09	0.59	0.43292	-0.45
NEG	L-Erythrulose	649.64	119.03	9.02	8.62	0.58	0.35607	0.07
NEG	Erucic acid	39.39	337.31	1.04	0.76	0.57	0.34210	0.46
NEG	Cytidine monophosphate	454.19	322.04	0.29	0.17	0.57	0.01051	0.77
NEG	Ethyl oleate	38.88	309.28	6.03	3.48	1.22	0.05857	0.79
NEG	Pyruvic acid	141.54	87.01	66.00	57.87	1.23	0.06969	0.19
NEG	Uracil	100.13	111.02	5.45	7.23	1.41	0.08381	-0.41
NEG	N-Acetylserine	320.15	146.04	0.82	1.05	1.50	0.08823	-0.35
NEG	Inosine	238.29	267.07	33.01	26.86	1.51	0.08983	0.30
NEG	Adenine	185.97	134.05	1.53	1.05	1.29	0.10564	0.54

NEG	(10E,12Z)-(9S)-9-Hydroperoxyoctadeca-10,12-dienoic acid	61.94	311.22	0.09	0.06	1.02	0.12475	0.43
NEG	Guanosine	288.14	282.08	1.55	1.23	1.03	0.14902	0.33
NEG	Adrenic acid	38.94	331.26	4.88	5.88	1.19	0.15079	-0.27
NEG	Nervonic acid	39.23	365.34	0.58	0.90	1.21	0.15096	-0.63
NEG	L-Allothreonine	389.27	118.05	0.75	1.08	1.17	0.16395	-0.53
NEG	L-Proline	334.86	114.05	4.23	5.60	1.79	0.17160	-0.41
NEG	Xanthine	235.68	151.02	6.34	8.57	1.55	0.22235	-0.43
NEG	Xanthosine	333.95	283.07	0.04	0.06	1.43	0.40572	-0.41
NEG	L-Norleucine	303.51	130.09	9.51	10.77	1.31	0.54940	-0.18
NEG	L-Phenylalanine	288.87	164.07	7.02	7.79	1.12	0.69579	-0.15
NEG	L-Valine	327.76	116.07	7.00	7.59	1.19	0.73457	-0.12
POS	Glycerylphosphorylethanolamine	415.21	216.06	1.12	0.36	0.99	0.01044	1.65
POS	Cymorcin diglucoside	451.89	491.21	0.02	0.01	0.99	0.00205	1.22
POS	PS(18:0/22:6(4Z,7Z,10Z,13Z,16Z,19Z))	220.79	836.54	0.17	0.21	0.99	0.14872	-0.34
POS	Phenylalanyl-Isoleucine	197.39	279.17	0.04	0.06	0.98	0.39026	-0.50
POS	LysoPC(18:3(6Z,9Z,12Z))	224.56	518.32	0.28	0.19	0.97	0.03527	0.57
POS	LysoPE(16:0/0:0)	229.96	454.29	5.74	1.74	0.97	0.11692	1.72
POS	Citrulline	418.75	176.10	0.28	0.48	0.97	0.18212	-0.79
POS	Persicaxanthin	37.18	385.27	0.27	0.32	0.95	0.37685	-0.26
POS	PC(20:2(11Z,14Z)/15:0)	173.07	772.58	0.99	0.82	0.95	0.32423	0.27
POS	L-Valine	294.92	118.09	9.07	11.65	0.95	0.11057	-0.36
POS	Isoniazid alpha-ketoglutaric acid	410.27	266.08	0.07	0.04	0.94	0.01044	0.76
POS	Butyrylcarnitine	283.73	232.15	9.57	6.75	0.94	0.04029	0.50
POS	PS(18:2(9Z,12Z)/18:0)	224.45	788.54	0.19	0.25	0.93	0.01037	-0.36
POS	Isoleucyl-Valine	219.84	231.17	0.06	0.08	0.92	0.26027	-0.42
POS	Diisopropyl sulfide	294.96	119.09	0.43	0.55	0.92	0.11162	-0.37
POS	alpha-Tocopherolquinone	22.47	447.38	0.01	0.01	0.91	0.13010	0.34
POS	L-2-Amino-5-hydroxypentanoic acid	50.86	134.08	0.28	0.38	0.91	0.23348	-0.44

POS	LysoPE(18:1(9Z)/0:0)	227.36	480.31	5.48	1.61	0.91	0.02871	1.77
POS	Oleamide	230.14	282.28	0.06	0.02	0.91	0.13016	1.81
POS	Glutaminylaspartic acid	453.37	262.10	0.08	0.08	0.91	0.98475	0.01
POS	1-Hydroxy-10-methylacridone	408.65	226.08	3.55	1.91	0.91	0.01839	0.89
POS	Guanine	287.52	152.06	1.46	1.22	0.90	0.22150	0.25
POS	PE(O-16:1(1Z)/22:6(4Z,7Z,10Z,13Z,16Z,19Z))	39.75	748.53	0.32	0.35	0.90	0.52027	-0.12
POS	Heptadecanoyl carnitine	204.04	414.36	0.14	0.22	0.90	0.11767	-0.71
POS	[12]-Gingerol	208.12	396.31	0.35	0.28	0.89	0.29129	0.31
POS	Molybdopterin precursor Z	482.91	364.06	0.03	0.02	0.89	0.01396	0.85
POS	Adenosine 3',5'-diphosphate	481.03	428.04	0.03	0.03	0.88	0.94929	-0.02
POS	Phosphocreatine	463.90	212.04	0.07	0.09	0.87	0.56970	-0.35
POS	Mangiferdesmethylursanone	32.50	429.37	0.62	0.81	0.87	0.05516	-0.38
POS	PS(18:1(9Z)/16:0)	225.55	762.53	0.08	0.08	0.86	0.66999	-0.09
POS	3-Dehydroxycarnitine	399.47	146.12	4.17	4.94	0.86	0.29802	-0.25
POS	PI(20:2(11Z,14Z)/18:2(9Z,12Z))	215.92	887.56	0.28	0.33	0.85	0.03564	-0.28
POS	Betaine aldehyde	331.80	102.09	1.01	0.73	0.84	0.16060	0.47
POS	Palmitic amide	34.38	256.26	0.06	0.06	0.83	0.15679	-0.18
POS	2-acetyl-1-alkyl-sn-glycero-3-phosphocholine	220.94	524.37	14.36	8.99	0.83	0.13978	0.68
POS	LysoPC(16:1(9Z)/0:0)	226.03	494.32	0.16	0.29	0.82	0.22621	-0.81
POS	LysoPE(0:0/18:3(6Z,9Z,12Z))	230.79	476.27	0.07	0.03	0.81	0.00382	1.11
POS	D-Alanine	52.27	90.06	0.30	0.51	0.80	0.07502	-0.80
POS	LysoPC(20:2(11Z,14Z))	220.67	548.37	0.18	0.21	0.80	0.65503	-0.18
POS	LysoPC(17:0)	222.59	510.36	0.61	0.32	0.80	0.09737	0.92
POS	hesperetin 3'-O-sulfate	419.70	383.04	0.02	0.02	0.79	0.29097	-0.20
POS	Racemethionine	313.51	150.06	1.80	1.84	0.78	0.95014	-0.03
POS	Cavipetin C	199.03	389.27	0.00	0.00	0.77	0.79696	0.14
POS	gamma-Glutamylglutamic acid	481.93	277.10	0.07	0.10	0.77	0.16592	-0.46
POS	PE(P-18:0/22:4(7Z,10Z,13Z,16Z))	164.18	780.59	0.23	0.30	0.76	0.17939	-0.38

POS	2-Methylbutyroylcarnitine	268.69	246.17	2.96	1.60	0.75	0.04204	0.89
POS	Propionylcarnitine	304.86	218.14	3.28	2.43	0.75	0.18638	0.43
POS	Homocysteine thiolactone	189.73	118.03	0.09	0.11	0.74	0.15309	-0.31
POS	1-Methyl-1,3-cyclohexadiene	36.71	95.09	0.05	0.07	0.74	0.01350	-0.49
POS	Deoxyguanosine	186.44	268.10	6.87	8.92	0.74	0.29998	-0.38
POS	PE(20:5(5Z,8Z,11Z,14Z,17Z)/P-18:0)	61.68	750.54	1.39	1.36	0.74	0.84195	0.03
POS	Adenosine 2'-phosphate	451.76	348.07	0.48	0.32	0.71	0.20426	0.57
POS	N-Ornithyl-L-taurine	241.80	240.10	0.05	0.03	0.71	0.19769	0.45
POS	L-Palmitoylcarnitine	205.70	400.34	5.37	6.69	0.71	0.21442	-0.32
POS	PI(20:2(11Z,14Z)/16:0)	220.94	863.56	0.04	0.04	0.71	0.64419	-0.09
POS	5-Methylcytidine	267.58	258.11	0.03	0.03	0.70	0.74679	-0.12
POS	Uridine	181.20	245.08	0.09	0.08	0.69	0.69341	0.09
POS	Valyl-Phenylalanine	209.50	265.15	0.04	0.03	0.68	0.78737	0.11
POS	(+)-2,3-Dihydro-3-methyl-1H-pyrrole	296.88	84.08	0.07	0.07	0.68	0.76714	-0.07
POS	6-Hydroxy-1H-indole-3-acetamide	47.95	191.08	0.06	0.04	0.67	0.15593	0.45
POS	Prolyl-Arginine	472.38	272.17	0.03	0.03	0.66	0.51887	-0.15
POS	Linoleamide	89.67	280.26	0.13	0.16	0.66	0.36383	-0.32
POS	PI(18:1(9Z)/18:1(9Z))	220.49	880.59	0.07	0.08	0.66	0.59915	-0.10
POS	Hypoxanthine	185.53	137.05	267.42	267.50	0.66	0.99711	0.00
POS	PC(22:5(7Z,10Z,13Z,16Z,19Z)/16:1(9Z))	167.76	806.57	2.18	2.18	0.65	0.96683	-0.01
POS	PC(P-16:0/18:4(6Z,9Z,12Z,15Z))	165.94	738.54	0.23	0.26	0.64	0.27358	-0.18
POS	DL-Glutamate	419.47	148.06	5.05	6.48	0.63	0.20513	-0.36
POS	3-Hydroxyisovalerylcarnitine	327.29	262.16	0.28	0.16	0.63	0.02530	0.81
POS	L-Arginine	443.26	175.12	0.36	0.42	0.62	0.15149	-0.21
POS	Citicoline	464.24	489.11	0.07	0.14	0.62	0.17285	-0.93
POS	1-Hydroxy-3-methyl-9H-carbazole	365.18	198.09	0.01	0.01	0.62	0.72691	0.10
POS	Octadecanamide	168.09	284.29	0.03	0.06	0.62	0.22982	-0.83
POS	Ethyl N-ethylanthranilate	198.73	194.12	0.09	0.05	0.62	0.48946	0.64

POS	PC(20:4(8Z,11Z,14Z,17Z)/15:0)	172.05	768.55	1.08	1.49	0.62	0.08000	-0.47
POS	2,5-Dihydro-2,4-dimethyloxazole	414.37	100.09	0.08	0.12	0.61	0.36360	-0.61
POS	LysoPI(18:0/0:0)	273.03	601.33	0.07	0.08	0.58	0.86978	-0.07
POS	Ecgonine	356.80	186.11	0.31	0.24	0.58	0.42467	0.33
POS	Threoninyl-Arginine	429.34	276.17	0.05	0.04	0.57	0.35509	0.26
POS	Taurine	318.06	126.02	21.56	23.05	0.57	0.40014	-0.10
POS	2-Methyl-5-(2-propenyl)pyrazine	239.95	135.09	0.07	0.09	0.57	0.12789	-0.26
POS	S-Adenosylhomocysteine	418.69	385.13	0.07	0.09	0.55	0.11523	-0.44
POS	PC(20:1(11Z)/14:0)	64.23	760.58	20.84	15.32	0.55	0.07234	0.44
POS	O-Phosphoethanolamine	488.55	142.03	4.53	4.91	0.55	0.51057	-0.11
POS	Alpha-dimorphecolic acid	36.24	279.23	0.10	0.10	0.55	0.54683	0.06
POS	LysoPE(0:0/20:4(5Z,8Z,11Z,14Z))	219.42	502.29	0.36	0.27	0.54	0.61876	0.43
POS	Alanyl-Arginine	438.08	246.16	0.29	0.34	0.54	0.42967	-0.21
POS	SM(d17:1/24:1(15Z))	204.95	799.67	0.04	0.05	0.54	0.30299	-0.30
POS	Cytidine monophosphate	454.04	324.06	0.12	0.07	0.53	0.01810	0.73
POS	Nervonyl carnitine	112.16	102.13	0.47	0.42	0.52	0.10951	0.14
POS	Norophthalmic acid	453.12	276.12	0.29	0.27	0.52	0.65349	0.13
POS	Zymonic acid	185.54	159.03	1.56	1.55	0.51	0.94303	0.01
POS	trans-Hexadec-2-enoyl carnitine	206.35	398.33	1.12	0.98	0.51	0.42104	0.19
POS	PC(P-18:1(11Z)/22:6(4Z,7Z,10Z,13Z,16Z,19Z))	162.32	816.59	0.03	0.03	0.51	0.42157	-0.30
POS	Prolyl-Threonine	350.16	217.12	0.02	0.01	0.51	0.07729	0.61
POS	Pisumionoside	423.22	405.21	0.25	0.30	0.51	0.28447	-0.25
POS	Glutaminyllysine	469.64	275.17	0.03	0.02	0.51	0.41359	0.20
POS	1H-Indole-3-carboxaldehyde	70.32	146.06	0.01	0.10	2.50	0.05119	-3.59
POS	beta-Alanine	389.05	90.06	0.30	0.38	1.64	0.05647	-0.35
POS	alpha-Terpinyl glucoside	419.94	317.19	0.01	0.01	1.28	0.05749	-1.10
POS	PC(22:4(7Z,10Z,13Z,16Z)/15:0)	170.40	796.58	0.27	0.33	1.16	0.06510	-0.28
POS	LysoPC(16:0)	224.49	496.34	29.15	14.82	1.01	0.06617	0.98

POS	3-Hydroxyadipic acid 3,6-lactone	329.03	145.05	0.12	0.11	1.36	0.06779	0.11
POS	PE(P-18:1(11Z)/22:4(7Z,10Z,13Z,16Z))	165.03	778.57	0.45	0.59	1.04	0.06800	-0.37
POS	PE(20:4(8Z,11Z,14Z,17Z)/P-16:0)	62.54	724.53	1.50	2.00	1.32	0.07125	-0.41
POS	Creatine	370.88	132.08	59.51	53.81	1.42	0.07850	0.15
POS	LysoPC(22:0)	216.23	580.43	0.05	0.03	1.16	0.07881	1.03
POS	Ergothioneine	351.09	230.09	0.01	0.02	1.26	0.08258	-2.02
POS	LysoPC(22:4(7Z,10Z,13Z,16Z))	218.23	572.37	0.03	0.04	1.47	0.08384	-0.67
POS	1-Kestose	473.40	527.16	0.02	0.01	1.01	0.08706	0.59
POS	3-Aminocaproic acid	38.09	132.10	0.37	0.53	1.11	0.08759	-0.52
POS	5-Aminopentanamide	304.14	117.10	0.20	0.14	1.04	0.09030	0.50
POS	Isoleucyl-Threonine	51.18	233.15	0.15	0.20	1.17	0.09829	-0.45
POS	D-Proline	333.96	116.07	14.37	19.37	1.91	0.10221	-0.43
POS	1-Methyl 2-galloylgalactarate	443.10	377.07	0.03	0.02	1.23	0.10486	0.24
POS	N-Methylalanine	48.62	104.07	0.48	0.71	1.25	0.10958	-0.55
POS	L-Asparagine	412.04	133.06	0.32	0.47	1.36	0.11323	-0.56
POS	PS(20:0/20:4(5Z,8Z,11Z,14Z))	221.62	840.57	0.10	0.15	1.17	0.11549	-0.52
POS	SM(d18:1/20:0)	207.27	759.64	0.11	0.09	1.76	0.13151	0.29
POS	Alanyl-Threonine	67.94	191.10	0.04	0.07	1.05	0.15056	-0.65
POS	PE(P-18:1(11Z)/16:0)	172.19	702.54	0.25	0.33	1.25	0.15610	-0.40
POS	Cytosine	218.95	112.05	0.40	0.57	1.04	0.15885	-0.51
POS	Inosine	238.15	269.09	5.00	4.29	1.38	0.16581	0.22
POS	1-(Hydroxymethyl)-5,5-dimethyl-2,4-imidazolidinedione	418.77	159.08	0.16	0.27	1.00	0.17678	-0.79
POS	Guanosine	287.53	284.10	0.81	0.67	1.04	0.18442	0.27
POS	1-Pyrroline	334.44	70.07	0.02	0.02	1.81	0.19607	-0.41
POS	Alanyl-Valine	45.37	189.12	0.16	0.20	1.14	0.20466	-0.38
POS	Dimethyl dialkyl ammonium chloride	151.37	304.30	0.06	0.02	1.25	0.22105	2.01
POS	2,3-Dihydro-5-(3-hydroxypropanoyl)-1H-pyrrolizine	37.08	180.10	0.24	0.32	1.07	0.23304	-0.43
POS	5-Aminopentanoic acid	407.87	118.09	0.58	1.01	1.64	0.24339	-0.79

POS	N-a-Acetyl-L-arginine	392.77	217.13	0.03	0.04	1.02	0.27515	-0.38
POS	LysoPC(P-16:0)	216.16	480.34	0.51	0.67	1.13	0.29620	-0.39
POS	Taurochenodeoxycholate-7-sulfate	263.81	530.27	0.01	0.01	1.21	0.30046	0.27
POS	Thiomorpholine 3-carboxylate	391.85	148.04	1.20	1.34	1.05	0.37369	-0.16
POS	Arachidonyl carnitine	197.83	504.40	0.02	0.02	1.19	0.37995	-0.41
POS	N6-Methyladenosine	308.76	282.12	0.17	0.23	1.47	0.38155	-0.45
POS	Thesinine 4'-O-glucoside	431.38	450.21	0.00	0.00	1.27	0.38622	0.44
POS	Ophthalmic acid	421.43	290.13	0.02	0.02	1.57	0.42180	0.27
POS	Piperidine	291.37	86.10	1.46	1.75	1.35	0.45981	-0.27
POS	3,3,5-triiodo-L-thyronine-beta-D-glucuronoside	291.90	132.10	9.49	11.27	1.36	0.46481	-0.25
POS	SM(d18:1/18:1(9Z))	207.30	729.59	0.61	0.56	1.38	0.46636	0.11
POS	D-Serine	409.73	106.05	0.18	0.23	1.44	0.52621	-0.32
POS	1-Methylguanosine	217.98	298.11	0.08	0.10	1.40	0.59223	-0.31
POS	Norvaline	326.36	118.09	5.35	5.92	1.14	0.62722	-0.15
POS	Pyrrolidine	326.94	72.08	0.10	0.12	1.26	0.62775	-0.19
POS	Ethyl 2-aminobenzoate	288.25	166.09	6.38	7.41	1.21	0.62898	-0.21
POS	(Â±)-erythro-Isoleucine	302.43	132.10	7.64	8.41	1.19	0.64007	-0.14
POS	N-gamma-L-Glutamyl-D-alanine	322.41	219.10	0.02	0.02	1.06	0.65642	-0.15
POS	7-Methylinosine	157.78	283.10	0.04	0.05	1.12	0.81728	-0.10
POS	N2,N2-Dimethylguanosine	213.81	312.13	0.04	0.05	1.13	0.83111	-0.17
POS	3'-O-Methylguanosine	252.92	298.11	0.01	0.01	1.71	0.99765	0.00
POS	Inosinic acid	468.34	349.05	0.05	0.02	1.00	0.03522	1.55

Note. rt: retention time; m/z: mass-to-charge ratio; VIP: variable importance in the projection; FC: fold change; GC:gastric cancer; PC: paracancerous; NEG: negative ion mode; POS: positive ion mode.

Table S2 Gene symbols related to metabolite pathways

SPTLC1	ACER1	PLPP3	GALC	CKMT1A	ALDH3A2	NOS3	DAO	PAICS	PNP	ENTPD8	GDA	ADCY4
SPTLC2	ACER3	PLPP2	GAL3ST1	CKMT2	ALDH1B1	ARG2	L3HYPDH	ADSL	HPRT1	ENTPD1	GUK1	ADCY5
SPTLC3	DEGS1	SGPP1	ARSA	CKB	ALDH7A1	ARG1	NUDT9	ATIC	IMPDH1	CANT1	PKM	ADCY6
KDSR	DEGS2	SGPP2	NEU1	CKMT1B	ALDH9A1	OAT	ADPRM	APRT	IMPDH2	ENTPD4	PKLR	ADCY7
CERS1	SGMS1	SPHK1	NEU3	AZIN2	CNDP1	PYCR3	NUDT5	NT5C2	NME6	ENTPD5	RRM1	ADCY8
CERS2	SGMS2	SPHK2	NEU4	AGMAT	CNDP2	PYCR2	PGM1	NT5C1A	NME7	ENTPD6	RRM2B	ADCY9
CERS4	SMPD1	SGPL1	NEU2	ODC1	CARNS1	PYCR1	PGM2	NT5C1B	NME2	NUDT16	RRM2	ADCY10
CERS3	SMPD2	UGCG	GLA	SRM	SAT2	PRODH	PRPS1L1	NT5C	NME4	ITPA	DGUOK	GUCY1A2
CERS6	SMPD3	GBA	PSAP	SMS	SAT1	ALDH18A1	PRPS2	NT5M	NME1	XDH	HDDC3	GUCY1A1
CERS5	SMPD4	GBA2	PSAPL1	AMD1	MAOB	LAP3	PRPS1	NT5C1B-RDH14	NME3	NUDT2	PRUNE1	GUCY1B1
ASAH1	ENPP7	B4GALT6	GATM	AOC1	MAOA	P4HA	PPAT	NT5E	NME1-NME2	GMPS	ADCY1	GUCY2C
ASAH2	CERK	GLB1	GAMT	SMOX	NOS1	PRODH2	GART	NT5C3A	AK9	GMPR	ADCY2	GUCY2D
ACER2	PLPP1	UGT8	CKM	ALDH2	NOS2	HOGA1	PFAS	NT5C3B	ENTPD3	GMPR2	ADCY3	GUCY2F
NPR1	PDE6G	ADA2	FHIT	COASY	GPD2	GNPAT	DGKG	PLA2G2E	PLA2G4D	PNPLA7	ETNPPL	MBOAT7
NPR2	PDE6H	AK7	ENPP4	AASDHPPT	GPAM	LPIN1	DGKQ	PLA2G3	PLA2G4F	GPCPD1	PEMT	GOT1

PDE1A	PDE9A	AK4	PAPSS	VNN1	GPAT2	LPIN3	DGKK	PLA2G2F	PLA2G6	CHAT	CDS1	GOT1L1
PDE1B	PDE10A	AK5	ENPP1_3	VNN2	GPAT4	LPIN2	CHPT1	PLA2G12A	PLB1	ACHE	CDS2	GOT2
PDE1C	PDE11A	AK2	PRHOXNB	VNN3	GPAT3	PLPP5	CEPT1	PLA2G12B	PLAAT3	CHKA	PLA1A	TAT
PDE2A	ADSS1	AK1	ALLC	BCAT2	AGPAT1	PLPP4	PLD1	PLA2G1B	LPCAT2	CHKB	PTDSS1	IL4I1
PDE3A	ADSS2	AK6	PANK1	BCAT1	AGPAT2	DGKZ	PLD2	PLA2G5	LPCAT1	PHOSPHO1	PTDSS2	PAH
PDE3B	AMPD2	AK3	PANK3	DPYD	AGPAT3	DGKD	PLD3	PLA2G2A	LPCAT4	PCYT1B	PISD	DDO
PDE5A	AMPD3	ENTPD2	PANK2	DPYS	AGPAT4	DGKI	PLD4	PLA2G2C	LPCAT3	PCYT1A	PGS1	ASRGL1
PDE6A	AMPD1	NTPCR	PPCS	UPB1	AGPAT5	DGKA	LCAT	PLA2G4E	LYPLA1	SELENOI	CRLS1	ASNS
PDE6B	ADK	PDE4	PPCDC	GADL1	LCLAT1	DGKE	PLA2G10	PLA2G4A	PLA2G15	ETNK1	TAZ	NIT2
PDE6C	DCK	PDE7	ENPP1	GPD1L	MBOAT1	DGKB	PLA2G2D	JMJD7-PLA2G4B	LYPLA2	ETNK2	LPGAT1	GPT2
PDE6D	ADA	PDE8	ENPP3	GPD1	MBOAT2	DGKH	PLA2G4C	PLA2G4B	PNPLA6	PCYT2	CDIPT	GPT
AGXT	AGXT2	ASS1	ASL	NAT8L	RIMKLB	RIMKLA	FOLH1	ASPA	GAD1	GAD2	ABAT	ALDH5A1
GLUD2	GLUD1	ALDH4A1	GLUL	CAD	GLS2	GLS	CPS1	GFPT2	GFPT1			
